# Exploring TRPC3 Interaction with Cholesterol through Coarse-Grained Molecular Dynamics Simulations

**DOI:** 10.3390/biom12070890

**Published:** 2022-06-25

**Authors:** Amy Clarke, Klaus Groschner, Thomas Stockner

**Affiliations:** 1 Institute of Pharmacology, Center for Physiology and Pharmacology, Medical University of Vienna, Waehringerstr., 13A, 1090 Vienna, Austria; amy.clarke@meduniwien.ac.at; 2 Gottfried Schatz Research Center, Division of Biophysics, Medical University of Graz, 8010 Graz, Austria; klaus.groschner@medunigraz.at

**Keywords:** cholesterol, TRPC3, molecular dynamics simulations, lipid–protein interactions, annular lipids

## Abstract

Transient receptor potential canonical 3 (TRPC3) channel belongs to the superfamily of transient receptor potential (TRP) channels which mediate Ca^2+^ influx into the cell. These channels constitute essential elements of cellular signalling and have been implicated in a wide range of diseases. TRPC3 is primarily gated by lipids and its surface expression has been shown to be dependent on cholesterol, yet a comprehensive exploration of its interaction with this lipid has thus far not emerged. Here, through 80 µs of coarse-grained molecular dynamics simulations, we show that cholesterol interacts with multiple elements of the transmembrane machinery of TRPC3. Through our approach, we identify an annular binding site for cholesterol on the pre-S1 helix and a non-annular site at the interface between the voltage-sensor-like domain and pore domains. Here, cholesterol interacts with exposed polar residues and possibly acts to stabilise the domain interface.

## 1. Introduction

The cellular plasma membrane is composed of membrane proteins embedded in a hydrophobic bilayer of hundreds of different types of lipids. These lipids provide a complex milieu for membrane proteins, with lipid composition determining not only the biophysical properties of the surrounding membrane but also the functional and multimerisation states of membrane proteins [1,2,3]. Lipids bound within the inner shell of a membrane protein can be classified as either annular or non-annular lipids. Annular lipids bind to the surface of membrane proteins, where they readily exchange with bulk lipids, but nonetheless maintain proteins in a folded state by providing them with the correct amphiphilic environment [4,5]. In contrast, non-annular lipids are typically bound at interfaces of subunits or at junctions between α helices, where they show slow exchange with bulk lipids. Non-annular lipids have diverse roles, including stabilising subunit interfaces, acting as allosteric modulators or regulating the function of membrane proteins [3,6].

The unique local lipid composition surrounding a membrane protein, including lipids present in annular and non-annular roles, has been termed the “lipid fingerprint” of a protein [7]. It has been shown through coarse-grained molecular dynamics (CG MD) simulations that different membrane proteins have distinct lipid fingerprints, an observation supported by analysis of protein–lipid interactions using mass spectrometry [8,9]. Coarse-grained molecular dynamics is a powerful tool for investigating this phenomenon, capable of achieving timescales that would be beyond the scope of all atom simulations, whilst still faithfully reproducing lipid-binding patterns observed in experimentally determined structures [10]. Through the use of CG MD, it has been shown that even members of the same family of transporters can have distinct lipid fingerprints, with knock-on effects for the local membrane biophysical properties [11]. Moreover, CG MD can distinguish between annular and non-annular lipids, whereas typical structure determination methods such as X-ray crystallography are limited in their ability to identify non-annular sites and are unsuitable for identifying annular sites.

The transient receptor potential canonical (TRP) channels constitute a superfamily of Ca^2+^-permeable ion channels, which are widely expressed in humans, including in neuronal cells, smooth muscle cells and cardiac cells [12,13,14]. The cryo-EM revolution has led to a recent boom in TRP channel structures, facilitating molecular dynamics explorations of their structure and dynamics, including possible mechanisms of activation. The transient receptor potential canonical 3 (TRPC3) channel is a member of the canonical subfamily of TRP channels and is primarily gated by diacylglycerol (DAG) [15]. In addition, studies have proposed that its surface expression is dependent on the presence of cholesterol [16]. Using a methyl-β-cyclodextrin (MβCD) approach, in which cholesterol-saturated MβCD was used as a cholesterol donor, it could be shown that the addition of cholesterol to HEK-293 cells results in elevated conductance and surface expression of TRPC3 [16], while cholesterol depletion by MβCD abolishes TRPC6 currents [17]. Cryo-EM structures of TRPC3 identified two lipid binding sites, designated L1 and L2 [18]; however, the identities of the lipids resolved at these binding sites were not determined. A subsequent combined lipid photo-pharmacology and molecular dynamics approach has proposed L2 as the binding site for DAG [19,20]. Little is known about the other lipids which define the inner shell of TRPC3 and how these lipids affect the TRPC3 microenvironment, including lipids important for stabilisation or protein function.

Cholesterol is a sterol which plays an important role in regulating membrane biophysical properties, including membrane fluidity, thickness and the presence of lipid rafts [21]. Due to its abundance and unique biophysical properties, cholesterol interaction and regulation of a variety of ion channels across several families has been reported, including TRP channels [22,23]. However, predicting cholesterol binding sites on membrane proteins has proved challenging, with cholesterol binding motifs such as the cholesterol recognition amino acid consensus motif (CRAC) [24] shown to have poor predictive power [25,26,27]. Moreover, cholesterol can exert its effects on channels through both direct and indirect mechanisms [28]. For example, cholesterol has been proposed to regulate the activation of the nicotinic acetylcholine receptor through direct interactions with the channel [29]. In contrast, the potassium channel MthK is indirectly regulated by cholesterol, exhibiting reduced ion permeation rates in cholesterol-containing membranes due to the larger lateral pressure exerted by these membranes [30].

In this study, we use 80 µs of coarse-grained molecular dynamics simulations to investigate the interaction of TRPC3 with cholesterol. We show that cholesterol displays a clear pattern of interaction enrichment around the centre of the protein, independent of the phospholipid composition of the membrane, where it interacts with multiple elements of the TRPC3 transmembrane domain. Maximum occupancy and contact duration analysis allowed us to decompose this binding into residues that bind cholesterol via frequent, but short-lived, contacts, and those that bind cholesterol via longer-lived contacts with little exchange of bound molecules. From this we could identify a cholesterol binding site at the interface between the voltage-sensor-like domain (VSLD) and pore domain, where cholesterol interacts with a lipid-exposed threonine residue.

## 2. Materials and Methods

### 2.1. System Preparation

The closed state cryo-EM structure (PDB: 6cud) resolved by Fan et al. [18] was downloaded from the Protein Data Bank [31] and Modeller 9.24 [32] was used to model missing residues and atoms. The protein was truncated to residues 322–699 (according to isoform 3 numbering). After determining the orientation of the protein in the membrane with the Position of Proteins in Membranes server [33], TRPC3 was then converted to a coarse-grained representation using the version 2.4 of the martinize.py script. TRPC3 was placed in a box of size 15 × 15 × 10 nm and the insane.py script [34] was used to add lipids according to the concentrations in Table 1. The system was solvated, neutralised and 150 mM NaCl was added, in addition to 10% anti-freeze water molecules. Four repeats were conducted for each membrane composition. For each repeat, the lipid molecule starting positions were different.

### 2.2. Simulation Parameters

Simulations were run on the Vienna Scientific Cluster (VSC3) using GROMACS version 2020.1 [35,36] with the Martini 2.2 forcefield for proteins and Martini 2.0 forcefield for lipids [37,38,39]. The simulations were conducted at a pressure of 1 bar, maintained using the Parrinello-Rahman barostat [40,41] and at a temperature of 310 K, maintained using a velocity rescaling thermostat [42]. The reaction-field algorithm [43] was used for electrostatic interactions with a cut-off of 1.1 nm, and a single cut-off of 1.2 nm was used for van der Waals interactions. The time step was 20 fs, with coordinates saved every 5000 steps. Neighbour searching was performed every 20 steps. During the production run, position restraints with a force constant of 100 were used to maintain the three-dimensional structure of TRPC3.

### 2.3. Analysis

Analysis was carried out using GROMACS version 2020.1 analysis tools, the GROmaρs package, VMD analysis scripts and the MDAnalysis module [36,44,45,46,47]. The 2D density maps were calculated using *gmx densmap*. GROmaρs was used to obtain an estimate of the absolute free energy of cholesterol binding, following the procedure described at (https://mptg-cbp.github.io/gromaps.html (accessed on 1 February 2022)) [44]. The remainder of the analysis was carried out using scripts written in-house, either using GROMACS analysis modules, VMD analysis tools or MDAnalysis. A 0.6 nm cut-off has been used throughout the paper to define a contact. Visualisation and image rendering were carried out using either Pymol version 2.3.0 (Schrödinger LLC, New York, NY, USA) or VMD version 1.9.4 [45].

## 3. Results

**Figure 1:**
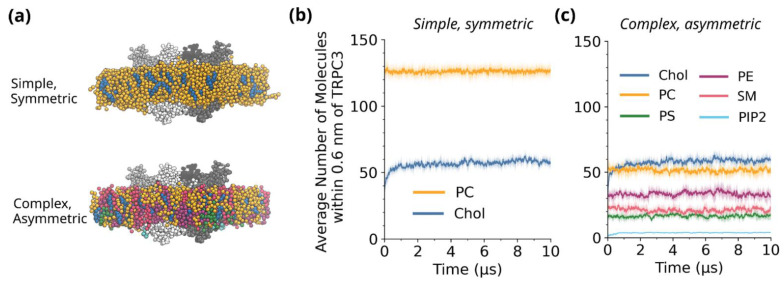
(**a**) Representative examples of the two membranes used in this study, a two-component symmetric membrane (**top**) and a six-component asymmetric membrane (**bottom**). The TRPC3 homotetramer is shown in greyscale, whilst the lipids are coloured according to the scheme in (**b**,**c**). (**b**,**c**) Number of lipid molecules within 0.6 nm of the TRPC3 homotetramer as a function of time, for the symmetric (**b**) and asymmetric (**c**) membranes. In dark is the mean averaged across four repeats; in light is the standard deviation. A window average of 20 ns has been applied.

In order to investigate the interaction of TRPC3 with its lipid environment, we simulated the protein in two types of coarse-grained membranes. System 1 is a two-component, symmetric membrane composed of 70% phosphatidylcholine (PC) and 30% cholesterol (Chol) (Figure 1a). System 2 is a six-component, asymmetric membrane composed of PC, Chol, phosphatidylethanolamine (PE), phosphatidylserine (PS), sphingomyelin (SM) and phosphatidylinositol bisphosphate (PIP_2_) and is designed to represent a simplified plasma membrane (Table 1) [48]. Four independent repeats in which the lipids were randomly placed in their starting configurations were run for each system, giving a total of 80 µs of simulation time. Prior to simulation, the lipids were randomly placed in a grid-like fashion around the protein, and during the course of the 10 µs-long simulations, the lipids could freely diffuse and interact with TRPC3. To begin, we calculated the average number of lipid molecules within 0.6 nm of the TRPC3 homotetramer as a function of time to quantify the lipid content in the first layer of lipids surrounding TRPC3 (Figure 1b,c). This allows us to determine when the protein and lipids have reached an equilibrium, and from this, it is clear that the phospholipids and cholesterol equilibrate rapidly in coarse-grained simulations with TRPC3, within a timescale of 1 µs. All further calculations are based on the equilibrated portion of the simulation (1 to 10 µs).

**Figure 2:**
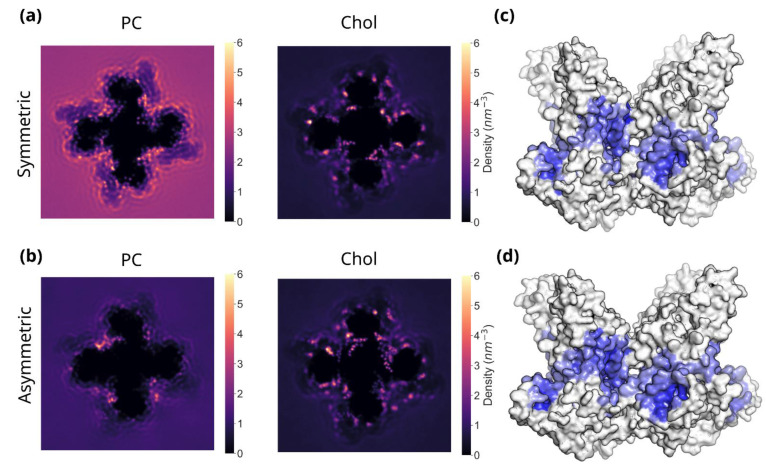
(**a**) Two-dimensional density maps showing PC and cholesterol densities for the two-component, symmetric membrane. For each system, the maps are averaged over four repeats and a Gaussian filter with a sigma value of 3 has been applied. (**b**) As in (**a**), for the six-component, asymmetric membrane. (**c**) Cholesterol enrichment map for the symmetric membrane, indicating in blue the residues where cholesterol binding is enriched (i.e., cholesterol accounts for over 30% of the contacts). Residues in grey indicate residues where cholesterol binding is not enriched. (**d**) As in (**c**), for the six-component, asymmetric membrane.

In the symmetric PC/Chol membranes, approximately 50–60 cholesterol molecules associate with TRPC3. Interestingly, when the complexity of the membrane is increased, the number of cholesterol molecules associated with the channel also plateaus at around 50–60 molecules per tetramer. Although cholesterol comprises 30% of both the symmetric and asymmetric membranes, this suggests that cholesterol binding to TRPC3 occurs independently of the phospholipid composition of the membrane, potentially due to the presence of buried cholesterol binding sites that are inaccessible or unfavourable to phospholipids. In contrast, the introduction of additional phospholipid species results in a clear decrease in the number of PC molecules bound to TRPC3 during the course of the 10 µs simulation. Two-dimensional density maps (Figure 2a,b) confirm the reduction in PC binding to TRPC3 in the presence of additional phospholipids, although areas of high PC density remain, possibly representing higher-affinity PC binding sites. Conversely, in both the symmetric and asymmetric membranes, multiple regions of high cholesterol density, clearly distinguishable from the bulk, can be observed.

In order to localise the regions of high cholesterol density to specific residues of the protein, the total number of contacts between each residue of TRPC3 and every lipid type of the system was calculated, where a contact is defined as occurring when two coarse-grained beads come within 0.6 nm of each other. The entire phospholipid/cholesterol (headgroups and tails) molecule was considered. From this it is possible to identify which lipid is most frequently in contact with the lipid-exposed residues of TRPC3, and also to identify residues where cholesterol binding is enriched (i.e., cholesterol accounts for over 30% of the contacts). Figure 2c,d shows the results of this. Both the symmetric and asymmetric membranes show a clear, consistent band of cholesterol enrichment around the centre of the protein, with cholesterol interactions occurring with multiple elements of the TRPC3 transmembrane machinery, including the pre-S1 helices and the VSLD. This is consistent with the analysis from Figure 1, which shows that cholesterol binding to TRPC3 occurs independently of the phospholipid composition of the membrane.

**Figure 3:**
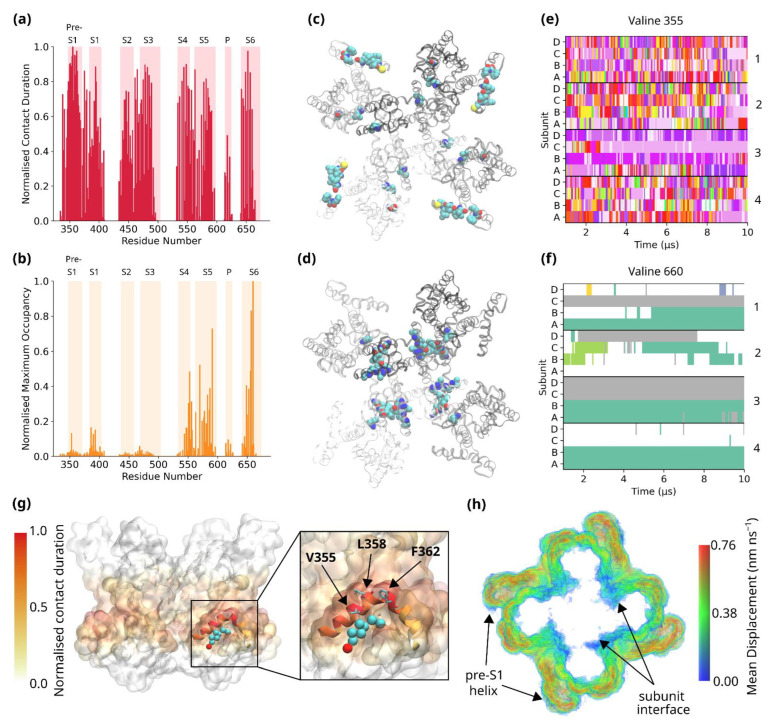
(**a**) Normalised contact duration values for TRPC3 interaction with cholesterol. The values are normalised with respect to Val-355, the residue with the highest average contact duration. All values are averaged across 16 monomers. The transmembrane helices of TRPC3 are indicated in light pink. (**b**) Normalised maximum occupancy values for TRPC3 interaction with cholesterol. The values are normalised with respect to Val-660, the residue with the greatest maximum occupancy value. All values are averaged across 16 monomers. The transmembrane helices of TRPC3 are indicated in light orange. (**c**) The ten residues of TRPC3 with the highest contact duration values, shown in cyan (carbon), red (oxygen), blue (nitrogen) and sulphur (yellow). The monomers of TRPC3 are shown in greyscale. (**d**) As in (**c**), for maximum occupancy. (**e**) Eventplot showing cholesterol interaction with Val-355. Every cholesterol binding event is represented by a different colour. (**f**) Eventplot showing cholesterol interaction with Val-660. (**g**) The three-dimensional structure of TRPC3, with residues coloured according to the contact duration values in (**a**). The pre-S1 helix is shown in cartoon representation, with a cholesterol bound under its arch-like structure. In red is the hydroxyl bead of Chol, in cyan are the remaining sterol and hydrocarbon tail beads. The zoom-in view highlights the hydrophobic residues on the pre-S1 helix. (**h**) Displacement map showing the mean displacement (nm ns^−1^) of cholesterol molecules within 0.6 nm of TRPC3.

Cholesterol enrichment at these residues could be due to the presence of long-term, stably bound cholesterol molecules or short-lived but frequent contacts. To distinguish between these two types of interactions, we applied the maximum occupancy and contact duration criteria devised by *Barbera N.* et al. [49] to our simulations. Maximum occupancy represents the longest continuous contact between a residue and a specific lipid molecule, and contact duration represents the total contact time between a protein residue and a specific lipid type. From the normalised contact duration (Figure 3a,c), it is clear that many of the lipid-exposed residues of TRPC3 make frequent contact with cholesterol molecules, resulting in relatively high contact duration values. The highest value corresponds to Val-355, with an average contact duration of 8624 ± 174 ns (average across 16 monomers). However, the corresponding maximum occupancy values suggest that many of these contacts are short-lived, with this analysis showing a markedly different pattern to the contact duration (Figure 3b).

Comparing the residues with the ten greatest contact duration values and those with the ten greatest maximum occupancy values shows that these residues are localised to very different regions of TRPC3 (Supplementary Table S1). The residues with the greatest contact duration values are largely hydrophobic residues found on the pre-S1 helix of TRPC3, including Val-355, Leu-358 and Phe-362. Here, the cholesterol molecules appear to dynamically exchange over the course of the simulation, with Val-355 interacting with over 100 different cholesterol molecules during the 10 µs simulation (Figure 3e). Cholesterol typically binds for tens of nanoseconds before unbinding. This likely reflects the nature of the pre-S1 helix, in a highly lipid-exposed region of the protein with an unusual elbow-like topology, which results in it penetrating only midway into the bilayer (Figure 3g) [18]. Consequently, a small hydrophobic cavity is formed under the arch of the elbow, where cholesterol binds but exchanges rapidly with bulk cholesterol molecules. This dynamic exchange is typical of annular lipid binding sites on membrane proteins [4,50]. Displacement analysis confirms the rapid exchange of cholesterol molecules at this site (Figure 3h). However, interestingly, it also shows that cholesterol molecules which bind to other regions of the VSLD are rapidly displaced, in agreement with the contact duration data. Taken together, they suggest that TRPC3 has a hydrophobic core capable of binding multiple cholesterol molecules, corresponding to the band identified in the enrichment calculations (Figure 2e–f), which are rapidly exchanged with bulk cholesterol molecules. The case of the pre-S1 domain is particularly interesting because of the topology of the pre-S1 helix (Figure 3g). It is possible that the preference of this site for cholesterol stems from reasons of shape complementarity. Although the hydrophobic residues of the pre-S1 helix could form interactions with the acyl chains of phospholipids, only cholesterol can be accommodated under the arch-like structure without significant entropic cost, due to its size.

**Figure 4:**
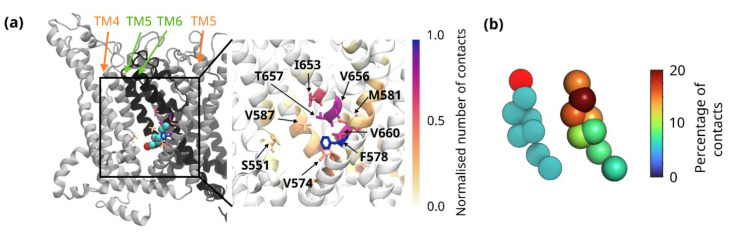
(**a**) TRPC3 shown in cartoon representation, where the residues in the stick representation are coloured according to the number of contacts made with cholesterol, once it has bound to Val-660. The left panel shows an example cholesterol molecule present at this site, taken from the final frame of a 10 μs simulation. The polar ROH group is coloured red. To highlight the subunit interface, one monomer is shown in black and the second monomer is shown in grey. In the zoom-in panel on the right, both monomers are shown in white. (**b**) The distribution of contacts between Thr-657 and bound cholesterol molecules. On the left is the coarse-grained representation of cholesterol, with the polar ROH group highlighted in red. The right shows the cholesterol beads coloured by the percentage of contacts they account for.

Conversely, the residues with the greatest maximum occupancy values are largely localised to the domain-swapped interface of the VSLD and pore domains, where displacement analysis shows slow diffusion of cholesterol molecules (Figure 3b,d,h). The residue with the highest maximum occupancy value, Val-660, is found on the S6 helix in a position that faces not only the bilayer but also the S5 helix of the adjacent subunit. Other residues identified through the maximum occupancy criteria include residues of the S4 (e.g., Ile-553) and S5 helices (e.g., Phe-591). These residues are found in a more buried location than those identified through contact duration. The hydrophobicity of cholesterol, which allows it to flip between leaflets of the bilayer without the requirement of a lipid transporter, likely facilitates its binding to these residues. Once bound, there is little exchange of cholesterol molecules, with Val-660 as a prime example that typically interacts with only one or two cholesterol molecules (Figure 3f), although not all Val-660 residues engage with a cholesterol during the course of the 10 µs simulation. Both the localisation of this residue to a domain interface and the slow exchange of cholesterol molecules are indicative of a non-annular binding site. In order to characterise this potential non-annular binding site better, we determined which residues are also in contact with cholesterol when it is bound to Val-660. As Figure 4a shows, cholesterol bound to Val-660 makes contacts with multiple residues at the interface between the VSLD and pore domain. Many of these residues, for example Thr-657 and Val-587, were also identified through the maximum occupancy analysis (Figure 3d). It is notable that this oligomerisation interface, as resolved by cryo-EM, has a large hollow towards the lower end of the transmembrane helices. It is possible that this hollow is occupied by cholesterol molecules that were not resolved in the cryo-EM structure. Interestingly, our data also show that cholesterol bound here still explores significant conformational space once bound, interacting with multiple residues from both domains (Figure 4a). This is in agreement with previous molecular dynamics simulations which have proposed that cholesterol rarely binds in a rigid manner to binding sites, preferring to bind to “greasy hollows” of the protein in which it can still adopt a range of binding poses [51,52].

The cluster of residues at the domain interface identified through maximum occupancy criteria are dominated by hydrophobic amino acids such as valine and leucine (Supplementary Table S1). However, cholesterol has a polar hydroxyl group attached to the sterol rings. The CRAC motif involves the stabilisation of this hydroxyl group through an interaction with either arginine or lysine. As there is no arginine or lysine present at the interfacial site of TRPC3, we hypothesised that the hydroxyl group of cholesterol may instead interact with Thr-657. This residue is localised on the S6 in the centre of the hydrophobic bilayer and was identified through the maximum occupancy analysis. In order to investigate this, we calculated the ratio of interactions between the eight different CG beads of cholesterol and Thr-657 (Figure 4b). This shows that cholesterol typically interacts with Thr-657 via a combination of its hydroxyl bead and adjacent sterol beads. The distribution of contacts over multiple beads is consistent with the flexible manner with which cholesterol binds to this site.

**Figure 5:**
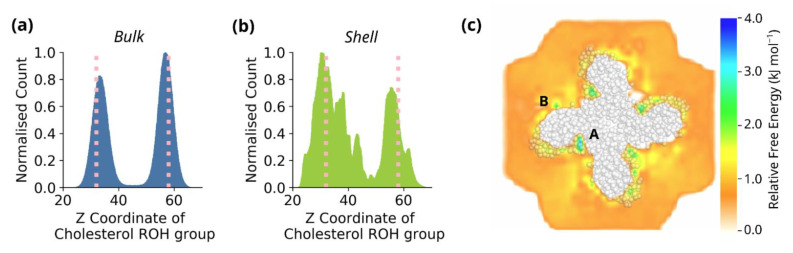
(**a**) The distribution of the ROH group along the *Z*-axis of the membrane for bulk cholesterol molecules. The pink dots present the Z coordinate of the PC glycerol groups. (**b**) As in (**a**), for cholesterol molecules bound to TRPC3. (**c**) Estimate of the absolute free energy of cholesterol binding to TRPC3, derived using GROmaρs, for a representative simulation. The energy is a relative scale with the minimum defined as 0. TRPC3 is displayed in grey spheres and the absolute free energy is displayed as a colourmap around the protein. The letters **A** and **B** refer to regions discussed in the text.

In order to compare the orientation of cholesterol bound to TRPC3 to the orientation of cholesterol in bulk lipids, we calculated the distribution of the ROH group of cholesterol along the Z axis of the membrane (Figure 5a). For cholesterol in bulk lipids, this shows a clear bimodal distribution, with a slight enrichment in the inner leaflet, and aligns with the Z distribution of the glycerol groups of PC. It appears reasonable to conclude from this that, with the exception of flipping events, cholesterol is typically oriented in the membrane with its polar hydroxyl group aligned with the glycerol groups of phospholipids and its hydrocarbon tail aligned with the hydrocarbon tails of phospholipids. Once bound to TRPC3, the distribution of the ROH group along the Z axis still assumes a largely bimodal distribution; however, there is now clear ROH density in the centre of the hydrophobic bilayer (Figure 5b). This can be attributed to cholesterol binding to buried sites, such as the interface between the pore and VSLD domains, where its hydroxyl group interacts with exposed polar residues.

Given the energetic cost of a polar amino acid localised to a lipid-exposed region in the centre of the hydrophobic bilayer and of the burial of the polar hydroxyl group of cholesterol, we calculated an estimate of the absolute free energy of cholesterol binding using GROmaρs [44]. This tool uses time-averaged density maps to determine spatial free energies, allowing us to estimate the absolute free energy of cholesterol binding in a specific region. Figure 5 shows that close to the protein are distinct regions of higher affinity compared to the bulk regions (Figure 5c). In particular, these regions can be localised to the interface between the pore domain and VSLD, where Val-660 and Thr-657 bind cholesterol in a potential non-annular binding site (region A, indicated in the figure), where, according to the relative scale, it is approximately 2–3 kJ mol^−1^ more favourable for a cholesterol to be bound than to be found in the bulk. This analysis suggests that the interfacial residues represent a high affinity binding site for cholesterol, allowing us to connect cholesterol binding in coarse-grained molecular dynamics simulations to free energy estimates. Interestingly, although the highest affinity sites are at the interface between the subunits, the GROmaρs analysis also identifies regions corresponding to the pre-S1 domain (region B, indicated in the figure). This suggests that free energy analysis can be applied to both annular and non-annular binding sites.

## 4. Discussion

The interaction of membrane proteins with their lipid environments is strikingly complex and multi-faceted, with recent computational work focusing on describing the individual “lipid fingerprints” of membrane proteins [7]. Although studies have investigated the role of DAG binding in TRPC3 channel activation [19], little is known about the roles of other lipids that comprise the TRPC3 annular and non-annular shell. TRPC3 surface expression has been shown to be dependent on the presence of cholesterol in membranes; however, a mechanism for this has not been delineated [16]. Deciphering the interaction of TRPC3 with its lipid environment is important not only for fine-tuning our understanding of this pharmacologically relevant channel [53], but also more broadly about how membrane proteins interact with their lipid shells [7].

In this study, we use a molecular dynamics approach to elucidate details of the TRPC3 interaction with cholesterol. Using 80 µs of coarse-grained simulation data, we applied the contact duration and maximum occupancy criteria to decompose the interactions into short-lived but frequent interactions, and longer-term, more stably bound interactions [49]. The results of this identify strikingly different residues. Contact duration highlights a cluster of residues on the pre-S1 helix where cholesterol molecules rapidly bind and unbind during the course of the simulation. This corresponds to a typical annular lipid binding site, where lipids are in fast exchange with bulk lipids [50]. The nature of this region likely facilitates cholesterol exchange: it is a highly lipid-exposed region, where the arch-like structure of the pre-S1 helix creates cavities which would be inaccessible to phospholipid tails. We propose that cholesterol preferentially binds to this region due to reasons of shape complementarity and steric inaccessibility to the acyl chains of phospholipids. Its presence here provides the correct amphiphilic environment for the unusual arch-like structure and it could be required for maintaining the structural integrity of TRPC3.

In contrast, maximum occupancy identified a binding site between the domain-swapped VSLD and pore domain, where cholesterol binds in a longer-term, more stably bound interaction. Both the localisation of this site and the length of cholesterol interaction here is suggestive of a non-annular binding site. Lipids bound to non-annular sites have been proposed to stabilise subunit interfaces and act in allosteric or regulatory capacities [3,9,25]. For example, molecular dynamics simulations have proposed that deeply buried, non-annular cholesterol stabilises the oligomeric nicotinic acetylcholine receptor by binding to gaps between the transmembrane helices of adjacent subunits [54]. In the absence of cholesterol, the secondary structure at the interface collapses in order to fill the gaps [54]. We propose that the binding of cholesterol to the non-annular site between the VSLD and pore domain could stabilise the tetrameric structure of TRPC3 in a similar way, particularly given the presence of a hollow towards the lower end of the transmembrane helices. However, it is worth noting that cholesterol does not occupy this site in all subunits of our simulations. Given the slow dynamics at the site, as confirmed by the displacement calculations, it is possible that longer timescales would be required to fully capture cholesterol occupancy and its dynamics.

Nonetheless, this binding site has several interesting features. Firstly, it is clear that cholesterol binds dynamically to this site, rather than binding in a rigid pose. This correlates with previously published molecular dynamics studies that have confirmed that cholesterol still exhibits significant flexibility even once bound to non-annular sites [25,52,55,56]. This has led to cholesterol binding sites being described as “greasy patches” or “greasy hollows”, in which cholesterol adopts a range of poses [51,52]. It has been proposed that this dynamic binding explains the lack of cholesterol binding sites identified through structure determination techniques such as X-ray crystallography. It has also proved to be a challenge for characterising cholesterol interactions via molecular dynamics simulations, with some studies opting for cut-offs as large as 1.0 nm for defining interactions between cholesterol and protein, in order to accommodate the flexibility of cholesterol binding [57]. Overall, 0.6 nm was chosen in our study as the cut-off in order to have a consistent definition between cholesterol and phospholipid interactions; however, other studies have variously defined contacts as 0.60, 0.63, 0.70 and 1.00 nm [7,49,56,57].

The second interesting feature of this site is that it does not correspond to the traditional cholesterol binding motif, CRAC, or its mirror, CARC. The CRAC motif is defined by the amino acid sequence (L/V)-X_1−5_-(Y)-X_1−5_-(K/R) and the CARC motif by the opposite sequence (K/R)-X_1−5_-(Y/F)-X_1−5_-(L/V) [24,58]. However, studies have questioned the predictive power of CRAC/CARC motifs for identifying cholesterol binding sites [25,26,27]. Instead, it has been proposed that these motifs represent a set of physicochemical principles underpinning cholesterol binding to proteins, with the hydroxyl group cholesterol typically hydrogen bonding to a polar amino acid and the hydrophobic portion of cholesterol interacting via van der Waals interactions to hydrophobic amino acids [27]. Consequently, our binding pose, consisting of threonine interaction with the hydrophilic moiety of cholesterol and hydrophobic residues interacting with the sterol and hydrocarbon tails of cholesterol, fits within this broader definition of a cholesterol binding site.

Previous molecular dynamics simulations investigating cholesterol–protein interactions have typically used simplified membranes consisting of PC/Chol [49,52,56]. In our study, we show that binding of cholesterol to TRPC3 occurs independently of the phospholipid composition of the membrane, with remarkable consistency between the enrichment patterns of the two-component symmetric membrane and the six-component asymmetric membrane. This finding validates approaches to exploring cholesterol interaction with membrane proteins that utilise simplified PC/Chol membranes, and also suggests that cholesterol binds to sites on transmembrane proteins that are either unfavourable or inaccessible to phospholipids. This observation has been previously proposed through a docking-based approach, and here we demonstrate this with extensive CG MD simulations of TRPC3 [59].

Two lipid binding sites on TRPC3 have been previously identified through cryo-EM structure determination [17]. The L1 binding site is localised to the inner leaflet in a cavity encircled by the pre-S1, S1 and S4 helices. Our predicted annular cholesterol binding site is found adjacent to the L1 binding site, in a cavity formed under the arch of the pre-S1 helix. Cholesterol binding here may be important for maintaining the structural integrity of the L1, although it is yet to be shown if the L1 is a regulatory or structural lipid binding site. The second binding site identified through cryo-EM, L2, is located between the pore and S6 helices of adjacent subunits. A combined electrophysiology and molecular dynamics approach has confirmed this as the DAG binding site [19]. Our proposed stable lipid binding site is located between the pore domain and VSLD, at a site in the inner leaflet, where it makes contacts with the S6, S5 and S4 helices of the domain-swapped homotetramer.

Our simulations provide a first molecular insight into the interaction between TRPC3 with cholesterol. We show that cholesterol binds to TRPC3 at the interface between the VSLD and pore domains, potentially stabilising the domain-swapped topology of the TRPC3 homotetramer. This represents an important step towards deciphering the cholesterol dependence of TRPC3 surface expression and more broadly how TRPC3 interacts with its lipid environment. Moreover, it endorses principles of cholesterol interaction with membrane proteins that have been derived from other molecular dynamics studies, including the dynamics of its binding, its range of interaction timescales and the independence of its binding from membrane phospholipid composition.

## Figures and Tables

**Table 1:** Membrane compositions used in this study. The abbreviations used in this table are phosphatidylcholine (PC), cholesterol (Chol), phosphatidylethanolamine (PE), phosphatidylserine (PS), sphingomyelin (SM) and phosphatidylinositol bisphosphate (PIP_2_).

Outer (%)	Inner (%)	Lipid
Simple, symmetric membrane		
70	70	PC
30	30	Chol
Complex, asymmetric membrane		
40	18	PC
30	30	Chol
25	10	PE
-	15	PS
20	10	SM
-	2	PIP2

## Data Availability

All raw data have been uploaded to zenodo.org (DOI:10.5281/zenodo.6579776 (accessed on 25 May 2022)).

## Supplementary Materials

The following supporting information can be downloaded at: https://www.mdpi.com/article/10.3390/biom12070890/s1. Table S1: Residues with the 10 greatest contact duration and maximum occupancy values.
