# Supplementary material for: Exploring TRPC3 Interaction with Cholesterol through Coarse-Grained Molecular Dynamics Simulations
Source: Biomolecules. 2022 Jun 25;12(7):890. doi: 10.3390/biom12070890 (PMC9313397; doi:10.3390/biom12070890)
Supplement: Supplementary file 1 [file biomolecules-12-00890-s001.zip › biomolecules-1768300-SI.pdf]

Supplementary Table S1. Residues with the 10 greatest contact duration and maximum occupancy values.

Rank	Maximum Occupancy	Contact Duration
1	Val-660	Val-355
2	Thr-657	Phe-362
3	Val-591	Ile-653
4	Leu-570	Leu-358
5	Val-656	Pro-361
6	Ile-553	Leu-354
7	Val-649	Leu-411
8	Val-587	Leu-548
9	Ile-653	Cys-350
10	Leu-583	Ser-478
